# YC-1 induces G_0_/G_1_ phase arrest and mitochondria-dependent apoptosis in cisplatin-resistant human oral cancer CAR cells

**DOI:** 10.1051/bmdcn/2017070205

**Published:** 2017-06-14

**Authors:** Miau-Rong Lee, Chingju Lin, Chi-Cheng Lu, Sheng-Chu Kuo, Je-Wei Tsao, Yu-Ning Juan, Hong-Yi Chiu, Fang-Yu Lee, Jai-Sing Yang, Fuu-Jen Tsai

**Affiliations:** 1 Department of Biochemistry, China Medical University Taichung 404 Taiwan; 2 Department of Physiology, China Medical University Taichung 404 Taiwan; 3 Department of Medical Research, China Medical University Hospital, China Medical University Taichung 404 Taiwan; 4 Chinese Medicinal Research and Development Center, China Medical University Hospital, China Medical University Taichung 404 Taiwan; 5 School of Pharmacy, China Medical University Taichung 404 Taiwan; 6 Department of Pharmacy, Buddhist Tzu Chi General Hospital Hualien 970 Taiwan; 7 Yung-Shin Pharmaceutical Industry Co., Ltd., Tachia Taichung 437 Taiwan; 8 Genetics Center, Department of Medical Research, China Medical University Hospital Taichung 404 Taiwan; 9 School of Chinese Medicine, China Medical University Taichung 404 Taiwan; 10 Department of Medical Genetics, China Medical University Hospital Taichung 404 Taiwan

**Keywords:** YC-1, G_0_/G_1_ phase arrest, mitochondria, Apoptosis, CAR cells

## Abstract

Oral cancer is a serious and fatal disease. Cisplatin is the first line of chemotherapeutic agent for oral cancer therapy. However, the development of drug resistance and severe side effects cause tremendous problems clinically. In this study, we investigated the pharmacologic mechanisms of YC-1 on cisplatin-resistant human oral cancer cell line, CAR. Our results indicated that YC-1 induced a concentration-dependent and time-dependent decrease in viability of CAR cells analyzed by MTT assay. Real-time image analysis of CAR cells by IncuCyte™ Kinetic Live Cell Imaging System demonstrated that YC-1 inhibited cell proliferation and reduced cell confluence in a time-dependent manner. Results from flow cytometric analysis revealed that YC-1 promoted G_0_/G_1_ phase arrest and provoked apoptosis in CAR cells. The effects of cell cycle arrest by YC-1 were further supported by up-regulation of p21 and down-regulation of cyclin A, D, E and CDK2 protein levels. TUNEL staining showed that YC-1 caused DNA fragmentation, a late stage feature of apoptosis. In addition, YC-1 increased the activities of caspase-9 and caspase-3, disrupted the mitochondrial membrane potential (AYm) and stimulated ROS production in CAR cells. The protein levels of cytochrome c, Bax and Bak were elevated while Bcl-2 protein expression was attenuated in YC-1-treated CAR cells. In summary, YC-1 suppressed the viability of cisplatin-resistant CAR cells through inhibiting cell proliferation, arresting cell cycle at G_0_/G_1_ phase and triggering mitochondria-mediated apoptosis. Our results provide evidences to support the potentially therapeutic application of YC-1 on fighting against drug resistant oral cancer in the future.

## 1. Introduction

According to the 2014 annual report of the Ministry of Health and Welfare, R.O.C. (Taiwan), cancer is the first leading cause of death among the ten leading chronic diseases in Taiwan. The number of cancer death reports was 46, 829 (28, 776 in men and 18, 053 in women), accounting for 28.6% of the total number of deaths. The death rate was 199.6 per 100, 000 population, increased by 1.3% from 2013 to 2014 [1, 2]. Oral cancer is the fifth leading cause of cancer death in Taiwan. The death rate of oral cancer was 11.4 per 100, 000 population [1, 2]. In Taiwan, the major risk factors of oral cancer are betel nut chewing [3–6], smoking [7], alcohol consumption [4, 8], inflammation [9, 10] and human papilloma virus (HPV) infection [11, 12]. The 5-year survival rate of oral cancer is 50% [13, 14]. Surgery, radiotherapy and chemotherapeutic drugs are the major treatments for oral cancer. The first-line chemotherapeutic drugs to treat oral cancer are cisplatin, carboplatin, 5-fluorouracil (5-FU), paclitaxel (Taxol®) and docetaxel (Taxotere®) [15–17]. However, surgery, radiotherapy and chemotherapy did not significantly improve the overall survival rate of oral cancer patients. On top of that, the development of drug resistance in the duration of chemotherapy remains as a clinical obstacle [18, 19]. To meet the need, designing novel compounds as well as discovering new targeting molecules that can overcome the resistance to chemotherapeutic drugs in oral cancer are clinically important.

YC-1 [3-(5’-hydroxymethyl-2’-furyl)-1-benzylindazole] was first designed and synthesized in our team [20, 21]. Current studies have shown that YC-1 has a wide spectrum of pharmacological activities, including anti-platelet [22–24], anti-inflammatory [25–29], anti-angiogenesis [30], neuro-protective [31–33], anti-hepatic fibrosis [34] and anti-cancer properties [20, 33, 35–56]. The underlying mechanism exerted by YC-1 included activation of NO-independent soluble guanylyl cyclase (sGC), inactivation of phosphodiesterase type 5 (PED5) [29, 57, 58] and inhibition of hypoxia-inducible factor 1a (HIF-1a) activity [22, 59, 60]. As for the anti-cancer activity, YC-1 can repress the proliferation of various types of cancer cells, including head and neck squamous cell carcinoma [48], esophageal squamous carcinoma [43], lung cancer [61–65], lymphoma [66, 67], bladder cancer [41, 68], hepatocellular carcinoma [52, 69], breast cancer [35, 55, 70], neuroblastoma [32], ovarian carcinoma [71], prostate cancer [72], pancreatic cancer [73], renal carcinoma [56, 74, 75], osteosarcoma [45], colon cancer [76, 77] and leukemia [20, 39, 49, 78]. In terms of the molecular mechanisms in anti-cancer activity, YC-1 induced cell cycle arrest at G_0_/G_1_ phase [50, 79, 80] or at S phase [52, 81], inhibited multidrug-resistant protein (MDR1) [82], reduced autophagy [83] and triggered apoptotic cell death [52]. In addition, YC-1 enhanced chemotherapeutic cisplatin sensitivity in hepatocellular carcinoma cells [84] and head and neck squamous cell carcinoma cells [48]. However, studies on whether YC-1 can inhibit cisplatin-resistant human oral cancer are scarce. The objective of this study was to investigate the anti-cancer effects of YC-1 on cisplatin-resistant human tongue squamous cell carcinoma CAR cells and its underlying mechanisms.

## 2. Material and methods

### 2.1. Chemicals and reagents

Cisplatin, propidium iodide (PI) and thiazolyl blue tetrazolium bromide (MTT) were purchased from Sigma-Aldrich (St. Louis, MO, USA). Trypsin-EDTA was purchased from BioConcept (Allschwil/BL, Switzerland). Fetal bovine serum (FBS), L-glutamine, penicillin G, 2’, 7’-dichlorodihydrofluorescein diacetate (H_2_DCFDA) and 3, 3-dihexyloxa-carbocyanine iodide [DiOC_6_(3)] were obtained from Thermo Fisher Scientific (Carlsbad, CA, USA). Caspase-3 and caspase-9 activity assay kits were purchased from R&D Systems Inc. (Minneapolis, MN, USA). The primary antibodies against Bcl-2, Bax, cytochrome *c*, Apaf-1, AIF, p21, cyclin A, cyclin D, cyclin E, CDK 2, β-actin and the goat anti-rabbit or anti-mouse IgG-horseradish peroxidase (HRP) secondary antibodies were purchased from GeneTex, (Hsinchu, Taiwan). Pan-caspase inhibitor (z-VAD-fmk) and enhanced chemiluminescence (ECL) detection kit (Immobilon Western Chemiluminescent HRP Substrate) were purchased from Merck Millipore (Billerica, MA, USA). YC-1 was designed and synthesized as detailed in the previous study [21].

### 2.2. Cell culture

The cisplatin-resistant cell line (CAR) was developed by treating CAL 27 cell line, a parental human tongue squamous cell carcinoma (American Type Culture Collection, Manassas, VA, USA) with 10-80 μM of cisplatin. CAR cells are characterized by its stable resistance to cisplatin as previously described [1, 18, 85, 86]. The cells were cultured in Dulbecco’s modified Eagle’s medium (DMEM) fortified with 10% fetal bovine serum (FBS), 100 U/ml penicillin, 100 μg/ml streptomycin, and 2 mM L-glutamine (Thermo Fisher Scientific) and were incubated at 37 °C with a humidified 5% CO2 air. The cisplatin-resistant CAR cells were constantly cultured in medium containing 80 μM cisplatin unless otherwise indicated [1, 18, 85, 86].

### 2.3. Cell viability assay

CAR cells (1 × 10^4^ cells/per well) were seeded in 96-well plates in 100 μl medium with or without 25, 50, 75 and 100 μM of YC-1 for 24 h. After YC-1 treatment, DMEM containing 500 μg/ml of MTT was added and incubated at 37 °C for 4 h. The medium was then removed, and 100 μl DMSO was added to each well to dissolve the formed blue formazan crystals, followed by measuring the 570 nm absorbance of each well by the ELISA plate reader with a reference wavelength of 620 nm. For the caspase inhibition experiment, cells were pretreated with 15 μM z-VAD-fmk (a pan-caspase inhibitor) for 1 h before subjected to YC-1 administration. Cell morphological examination was observed and photographed by the IncuCyte^™^ Kinetic Live Cell Imaging System (Essen BioScience, Ann Arbor, MI, USA) [87–89].

### 2.4. IncuCyte cell proliferation and confluence assay

To measure the cell confluence, a stable mixture of CAR cells (2 × 10^4^ cells) were plated into a 96-well plate. The cells were then incubated with or without 25, 50, 75 and 100 μM of YC-1. Cell confluence relative to the control cells was determined by the IncuCyte^™^ Kinetic Live Cell Imaging System (Essen BioScience) at a 2-h interval and up to 48 h [90].

### 2.5. Flow cytometry analysis of cell cycle distribution

CAR cells (2 × 10^5^ cells/per well) were plated into the 12-well plates and then treated with 100 μM of YC-1 for 0, 12, 24, 36 and 48 h. The cells were then fixed, followed by staining with propidium iodide (PI) solution as previously described [91, 92]. The cell cycle profiling and the data analysis were determined utilizing a Muse Cell Analyzer (Merck Millipore, Hayward, CA, USA) [93–98].

### 2.6. Immunoblotting analysis

CAR cells (1 × 10^7^/75-T flask) were treated with 0, 25, 50, 75 and 100 μM of YC-1 for 48 h. The cells were then harvested, and the total proteins in cell lysate were collected by SDS sample buffer. Briefly, protein sample from each treatment was subjected to electrophoresis on a 10% SDS-polyacrylamide gel (SDS-PAGE), followed by electro-transferring to a PVDF membrane. The transferred membranes were blocked in 20 mM Tris-buffered saline/0.05% Tween-20 solution containing 5% non-fat dry milk for 1 h at room temperature. The membrane was then probed with the primary antibodies against proteins associated with either cell cycle regulation or apoptosis at 4 °C overnight. Afterwards, the membranes were washed with Tris-buffered saline/Tween-20 and incubated with secondary antibodies conjugated with horseradish peroxidase (HRP). The blots were developed by an enhanced chemiluminescence kit (Immobilon Western HRP Substrate; Merck Millipore, Bedford, MA, USA), followed by X-ray film exposure [99, 100].

### 2.7. TUNEL staining

CAR cells (2 × 10^5^ cells/ per well) were seeded into 12-well plates and incubated with 0, 25, 50, 75 and 100 of YC-1 for 48 h. At the end of the treatment, apoptotic DNA fragmentation was detected using the *In Situ* Cell Death Detection kit, Fluorescein (Roche Diagnostics GmbH, Roche Applied Science, Mannheim, Germany) according to the protocol by the manufacturer [101–104].

### 2.8. Assays for caspase-3 and caspase-9 activities

CAR cells (2 × 10^5^ cells/ per well) were seeded into 6-well plates and incubated with 0, 25, 50, 75 and 100 of YC-1 for 48 h. At the end of the treatment, cells were harvested and cell lysates were assessed in accordance with the manufacturer’s instruction provided in the caspase-3 and caspase-9 Colorimetric Assay kits (R&D Systems Inc.). Cell lysate protein was then incubated for 1 h at 37 °C with specific caspase-3 substrate (DEVD-pNA) or caspase-9 substrate (LEHD-pNA) in the reaction buffer (provided in the kits). The OD_405_ of the released pNA in each sample was measured as previously described [86, 105].

### 2.9. Detection of ROS generation and mitochondrial membrane potential (ΔΨm)

CAR cells (2 × 10^5^ cells/ per well) were seeded into 6-well plates and incubated with 0, 25, 50, 75 and 100 of YC-1 for 48 h. At the end of the treatment, cells were harvested and incubated with 10 μM H_2_DCFDA and 4 nM DiOC_6_ at 37 °C for 30 min for H_2_O_2_ detection and A¥m, respectively. The mean fluorescence intensity (MFI) was quantified by BD CellQuest Pro software (BD Biosciences, San Jose, CA, USA) after analysis by flow cytometry [86, 105, 106].

### 2.10. Statistical analysis

All the statistical results are presented as the mean ± sd for at least three separate experiments. Statistical analysis of data was done using one-way ANOVA followed by Student’s t-test. ***P < 0.001 was considered statistically significant.

## 3. Results

### 3.1. YC-1 decreased the viability and suppressed confluence of CAR cells

**Fig. 1:**
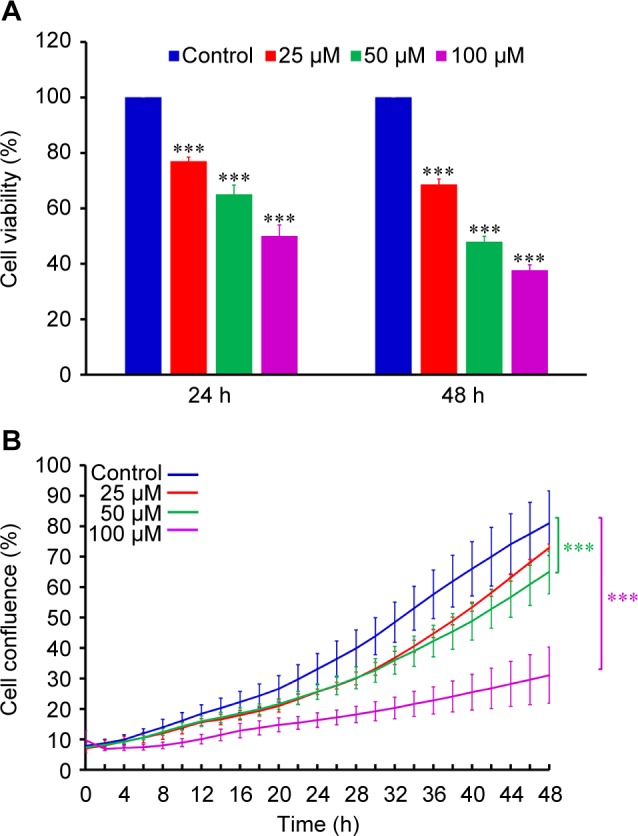
Effects of YC-1 on cell viability and cell confluence in CAR cells. Cells were incubated with 0, 25, 50 and 100 μM of YC-1 for various duration. (A) The cell viability was determined by MTT assay. (B) The cell confluence was determined by the IncuCyte™ Kinetic Live Cell Imaging System. Data are presented as the mean ± sd (n = 3). ***p < 0.001 versus untreated control.

**Fig. 2:**
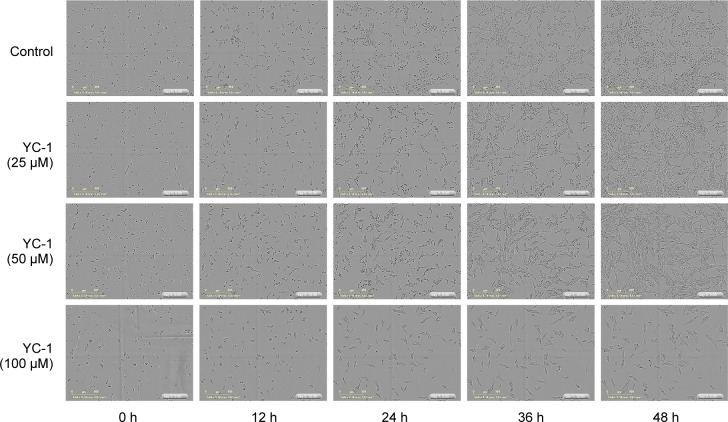
Effects of YC-1 on cell morphology and confluence of CAR cells. Cells were incubated with 0, 25, 50 and 100 of YC-1 for 0, 12, 24, 36 and 48 h. The cell morphology and density was determined by the IncuCyte™ Kinetic Live Cell Imaging System.

The cisplatin-resistant human oral CAR cells were treated with YC-1 (0, 25, 50 and 100 μM) for either 24 h or 48 h. The MTT assay demonstrated that YC-1 significantly decreased the cell viability in a concentration and time-dependent manner (Fig. 1A). The percentage of cell confluence relative to the control cells was determined by the IncuCyte™ Kinetic Live Cell Imaging System at a 2-h interval and up to 48 h. The administration of YC-1 (0, 25, 50 and 100 μM) inhibited the confluences of cultured CAR cells (Fig. 1B). The inhibition of cell confluence showed concentration and time-dependent. Images of cultured CAR cells under different YC-1 concentrations (0, 25, 50 and 100 μM) taken by IncuCyte^™^ Kinetic Live Cell Imaging System at the indicated period of time showed that YC-1 induced cell morphology changes and triggered cell death (Fig. 2). Herein, we also provide the realtime cell imaging of cultured CAR cells with or without YC-1 (100 μM) by IncuCyte™ Kinetic Live Cell Imaging System video (Supplementary video). Our data revealed that YC-1 exhibited cytotoxicity to CAR cells.

### 3.2. YC-1 caused G_0_/G_t_ cell cycle arrest and affected the expression levels of G_0_/G_1_ proteins of CAR cells

**Fig. 3:**
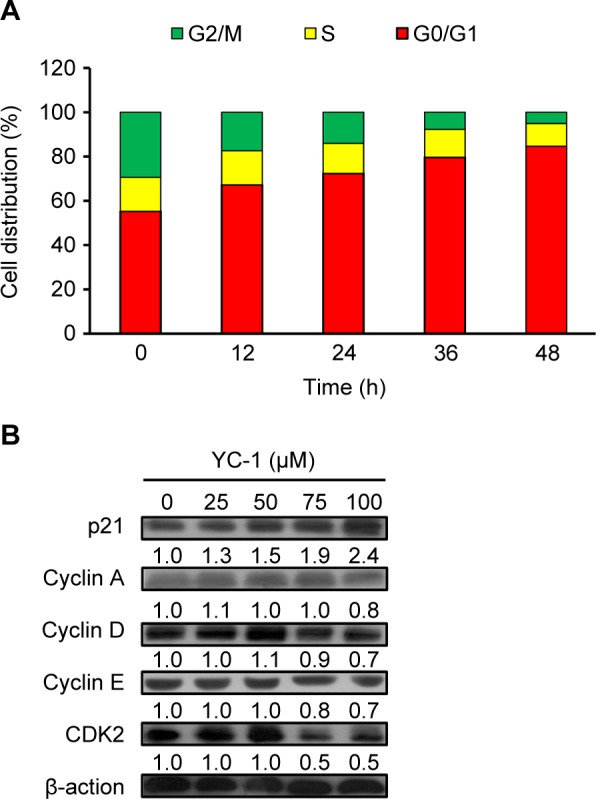
Effects of YC-1 on cell cycle distribution and the levels of G_0_/G_1_ proteins of CAR cells. (A) Cells were incubated with 100 μM of YC-1 for 0, 12, 24, 36 and 48 h. The cell cycle distribution was assessed by PI staining and flow cytometric analysis. (B) Whole-cell lysates were prepared, and the levels of G_0_/G_1_ proteins were analyzed by western blot analysis.

To verify whether YC-1 treatment affects the cell cycle distribution, CAR cells were administered with 100 μM of YC-1 for 0, 12, 24, 36 and 48 h. The percentage of cells in G_0_/G_1_, S and G_2_/ M phase were analyzed by DNA content stained with PI and flow cytometry. Our data indicated that YC-1 treatment resulted in cell cycle arrest at G_0_/Gj phase. The percentage of cells arrested at G_0_/Gj increased as the treatment duration lengthened. In the meanwhile, a marked decrease of the cells at G_2_/M phase was observed (Fig. 3A). The expression levels of proteins associated with G_0_/Gj were analyzed after 24-h treatment. YC-1 induced the protein expression of p21 in a concentration-dependent manner, while the protein expression of cyclins A, D, E and CDK2 was inhibited (Fig. 3B). These results indicated that YC-1 regulated CDK2 activation and caused G_0_/G_1_ phase arrest in the CAR cells.

### 3.3. YC-1 induced DNA fragmentation and enhanced cas- pase-9 and caspase-3 activities in CAR cells.

**Fig. 4:**
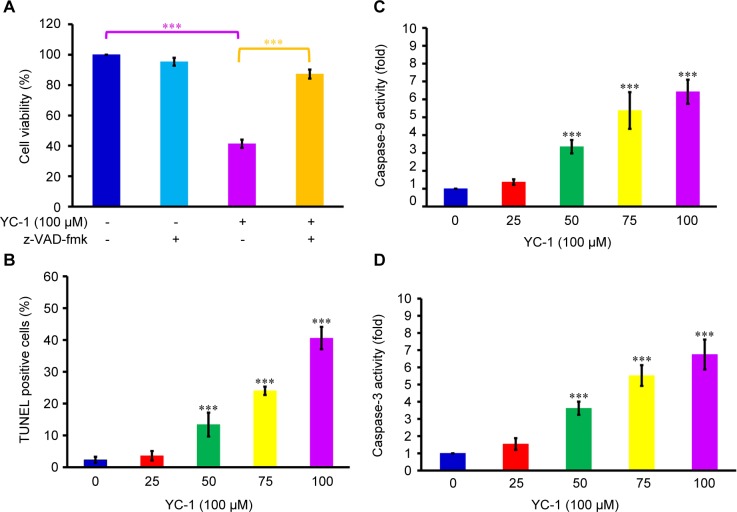
Effects of YC-1 on DNA fragmentation, caspase-9 and caspase-3 activities in CAR cells. (A) Cells were incubated with 100 μM of YC-1 with or without z-VAD-fmk for 48 h. The cell viability was determined by MTT assay. (B) TUNEL assay, (C) caspase-9 and (D) caspase-3 activities were analyzed in CAR cells treated with 0, 25, 50, 75 and 100 μM of YC-1 for 48 h. Data are presented as the mean ± sd (n = 3). ***P < 0.001 versus untreated control.

We examined whether YC-1 induces apoptosis in CAR cells. A significant reduction in cell viability from MTT assay was observed after cells were exposed to 100 μM of YC-1 for 48 h. However, the decreased cell viability induced by YC-1 was reversed by z-VAD-fmk (a pan-caspase inhibitor) (Fig. 4A). Results from TUNEL staining also showed that as the YC-1 concentration increased, more TUNEL positive cells were observed, indicating that more cells exhibited DNA fragmentation (Fig. 4B). To further investigate whether the cell death provoked by YC-1 was mediated through caspases activation, protein samples collected from CAR cells after YC-1 exposure for 48 h were analyzed. Treatment of YC-1 (0, 25, 50, 75 and 100 μM) significantly and concentration-dependently stimulated the activities of both caspases-9 and caspase-3 (Fig. 4C and 4D). Our data demonstrated that YC-1 induced apoptosis, and the activation of caspases was involved in apoptotic cell death in CAR cells.

### 3.4. YC-1 stimulated ROS production, collapsed mitochondrial membrane potential (AΨm) and altered the levels of apoptosis-related proteins in CAR cells

**Fig. 5:**
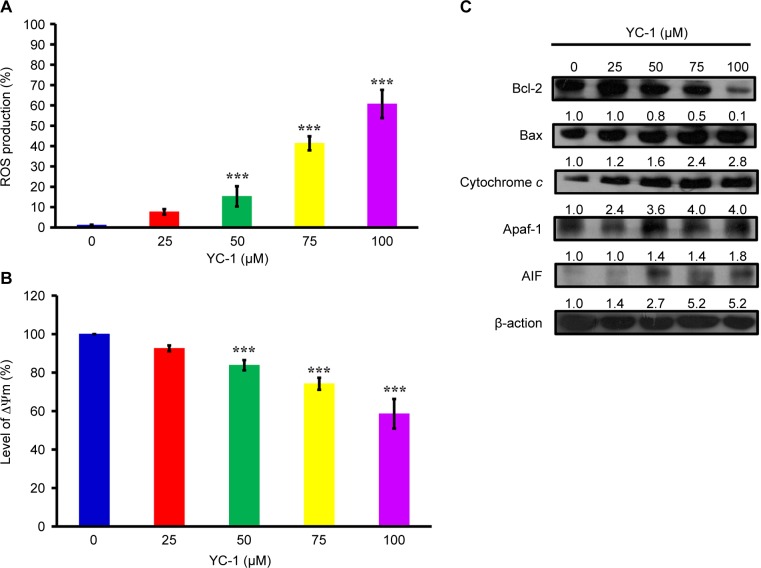
Effects of YC-1 on ROS, mitochondrial membrane potential (∆Ψm) and the levels of apoptosis-related proteins in CAR cells. Cells were incubated with 0, 25, 50, 75 and 100 of YC-1 for 48 h. (A) ROS level was assessed by staining with H_2_DCFDA, and (B) loss of A¥m was measured with DiOC_6_(3) by flow cytometry. Data are presented as the mean ± sd (n = 3). ***P < 0.001 versus untreated control. (C) Whole-cell lysates were prepared, and the levels of apoptosis related proteins were analyzed by western blot analysis Data is presented.

We investigated whether YC-1 stimulates ROS production. The production of ROS markedly elevated after cells were administrated with of YC-1 (0, 25, 50, 75 and 100 μM), and the elevation showed concentration-dependent (Fig. 5A). To confirm whether the mitochondrial pathway mediating YC-1-induced cell apoptosis, the level of ΔΨm was measured, and immunoblotting analysis was performed to evaluate the expression levels of proteins associated with mitochondria-dependent apoptotic pathways. CAR cells exhibited a decrease of AYm in a concentration-dependent manner after 48 h of YC-1 treatment (Fig. 5B). YC-1 suppressed the level of Bcl-2, while it promoted the protein expressions of Bax, cytochrome *c*, Apaqf-1 and AIF (Fig. 5C), indicating the involvement of mitochondria-dependent pathway.

## 4. Discussion

Discovering and exploring novel therapeutic strategy and underlying molecular mechanisms has been a major research focus in oral cancer therapy [107–110]. Studies on various cancer cells demonstrated that YC-1 possessed significant anti-cancer activities through several pathways. YC-1 can induce cell cycle arrest [81, 111, 112], apoptosis [81, 111, 112] and autophagy [83, 113, 114]. It also blocked angiogenesis [30, 115–117], cell migration [41, 43, 72, 118], metastasis [36, 64, 119] and reduce matrix metalloproteinases (MMPs) activity [41, 72, 117]. Furthermore, YC-1 enhanced the chemo-sensitivity of cancer cells to cisplatin by regulating expression and activity of apoptosis-related proteins, leading to the activation of caspase-9 and caspase-3 signaling [120]. Recently, Tuttle *et al.* [48] reported that YC-1 inhibited cell proliferation, induced apoptotic cell death, and increased sensitivity to cisplatin in UM-1- and CAL 27-cisplatin resistance cells. However, the molecular mechanisms of YC-1-induced cell cycle arrest and death in cisplatin resistant oral cancer cells are not yet fully understood. In this study, our results showed that 25-100 of YC-1 significantly inhibited the proliferation of cisplatin-resistant CAR cells (Fig. 1, Fig. 2 and Supplementary video). YC-1 treatment increased the number of cells in the G_0_/ G1 phase, suggesting that YC-1 caused growth inhibition by promoting G0/G1 phase arrest in CAR cells (Fig. 3). The significant DNA fragmentation and caspase-3/ -9 activation in YC-1 treated cells (Fig. 4B, C, and D) indicate that YC-1 can induce caspase- dependent apoptosis in CAR cells. Our findings provide new insights addressing the anti-cancer activity of YC-1 in cisplatin-resistant CAR cells at the molecular levels.

Once the mitochondrial apoptotic signaling is provoked, changes in the mitochondrial membrane permeability would lead to the loss of mitochondrial membrane potential. In addition, the mitochondrial outer membrane becomes leaky and releases the proapoptotic proteins; including cytochrome *c*, Apaf-1, procas- pase-9, AIF and Endo G into cytosol. These proteins can then activate caspase-9 and caspase-3 and result in DNA fragmentation, a unique feature of the late stage apoptosis [121–125]. Bcl-2 family proteins are also involved in the regulation of apoptosis through modulating mitochondrial functions [121, 124]. Our results showed that YC-1 induced apoptosis, as evidenced by the reduced viability and the significant number of TUNEL-positive cells (Fig 4A, B). YC-1 induced apoptosis was further confirmed by pan-caspase inhibitor which reversed the reduction of cellular viability in YC-1 treated cells (Fig 4A). In addition, the loss of ΔΨm, elevation of ROS production, and the changes in quantity of mitochondria-related proteins (Bcl-2, Bax, cytochrome *c,* Apaf-1 and AIF) were observed after YC-1 treatment (Fig. 5). These results suggested that YC-1-induced apoptosis was mediated through the activation of caspase cascades, and this apoptotic death was mitochondria-dependent. This study is the first report to prove the involvement of a mitochondrial pathway in YC-1-induced apoptosis in cisplatin-resistant CAR cells.

It has been documented that YC-1 inhibited cell proliferation and cell cycle progression from G_0_/G_1_ to S phase in rat mesangial cell and human hepatocellular carcinoma cells [50, 80]. Teng *et al.* [50] demonstrated that YC-1 inhibited human hepatocellular carcinoma cell proliferation through G_0_/G_1_ phase arrest and increased p21 and p27 protein levels. However, Yeo. *et al.* reported YC-1 induced S phase arrest and apoptosis in Hep3B cells [81]. Our results (Fig 3) were consistent with those of Teng *el al*. [50] and suggested that, by down-regulation of CDK2/cyclin A, D, and E activities, YC-1 blocked cell cycle at G_0_/G_1_ phase.

The IncuCyte™ Kinetic Live Cell Imaging System provides a continuous time-lapsed recording and quantitation of cell life images, which facilitates a robust data collection and analysis. This system can be used to detect cell activities such as cell proliferation, migration, invasion, wound healing, caspase activity and autophagy [126-128]. In the present study, we are the first group using this imaging system to characterize cell proliferation and confluence in YC-1-treated CAR cells (Fig. 2 and Supplementary video). Thus, more studies on anti-cancer activity of YC-1 can be accelerated and examined by this cell image system in the near future.

## 5. Conclusions

**Fig. 6:**
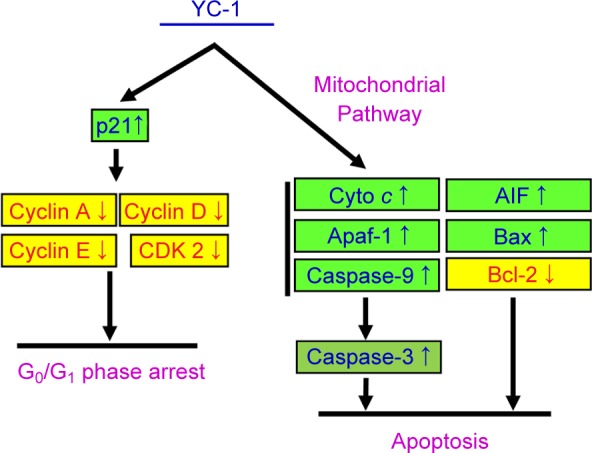
Schematic diagram of proposed molecular mechanism of YC-1-induced G_0_/G_1_ phase arrest and apoptosis in cisplatin- resistant human oral cancer CAR cells.

Fig. 6 illustrated the proposed molecular mechanism of YC-1-provoked G_0_/G_1_ phase arrest and apoptosis in CAR cells. Our results revealed that YC-1 arrested at G_0_/Gj phase through regulating p21, cyclin A, D, E and CDK2 activity. In addition, YC-1 induced apoptosis in CAR cells *via* caspases activation and mitochondria-dependent pathway. YC-1 is proved to be potential adjuvants or alternatives to cisplatin treatment in cisplatin-resistant oral cancer.

## Competing interests

The authors declare that they have no competing interests.

## Supplementary video

Effects of YC-1 on cell confluence in CAR cells. cclls were incubatcd with or without 100 μM of YC-1. The dyuamic ccll imaging was 3en by the IneuC’yte™ Kinetic Live Cell Imaging System at a 2 h interval and up to 48 h
